# Branched-Chain Amino Acid Catabolism and Cardiopulmonary Function Following Acute Maximal Exercise Testing in Adolescents

**DOI:** 10.3389/fcvm.2021.721354

**Published:** 2021-08-18

**Authors:** Pinar Gumus Balikcioglu, Megan E. Ramaker, Kelly A. Mason, Kim M. Huffman, Johanna L. Johnson, Olga Ilkayeva, Michael J. Muehlbauer, Michael Freemark, William E. Kraus

**Affiliations:** ^1^Division of Pediatric Endocrinology and Diabetes, Duke University School of Medicine, Durham, NC, United States; ^2^Duke Molecular Physiology Institute, Duke University School of Medicine, Durham, NC, United States; ^3^Division of Endocrinology, Metabolism, and Nutrition, Duke University School of Medicine, Durham, NC, United States; ^4^Division of Adult Cardiology, Duke University School of Medicine, Durham, NC, United States

**Keywords:** total body oxygen consumption (VO_2_), rVO_2_, growth hormone, ghrelin, BCAA

## Abstract

**Background:** To provide energy for cardiopulmonary function and maintenance of blood glucose, acute aerobic exercise induces lipolysis, fatty acid oxidation (FAO), glycolysis, and glycogenolysis/gluconeogenesis. These adaptations are mediated by increases in cortisol, growth hormone (GH), and catecholamines and facilitated by a decline in insulin. Branched-chain amino acids (BCAA) also undergo catabolism during intense exercise. Here, we investigated the relationship between BCAA catabolism and metrics of cardiopulmonary function in healthy, well-developed, mature adolescent athletes undergoing an acute bout of maximal aerobic exercise.

**Hypothesis:** We hypothesized: (a) acute maximal exercise in adolescents induces lipolysis, FAO, and BCAA catabolism associated with increases in GH and cortisol and a reduction in insulin; (b) increases in GH are associated with increases in ghrelin; and (c) metrics of cardiopulmonary function (aVO_2_, rVO_2_, aVO_2_/HRmax) following maximal exercise correlate with increases in GH secretion, FAO, and BCAA catabolism.

**Methods:** Blood samples before and after maximal cardiopulmonary exercise in 11 adolescent athletes were analyzed by tandem-mass spectrometry. Paired, two-tailed student's *t*-tests identified significant changes following exercise. Linear regression determined if pre-exercise metabolite levels, or changes in metabolite levels, were associated with aVO_2_, rVO_2_, and aVO_2_/HRmax. Sex and school of origin were included as covariates in all regression analyses.

**Results:** Following exercise there were increases in GH and cortisol, and decreases in ghrelin, but no changes in glucose or insulin concentrations. Suggesting increased lipolysis and FAO, the levels of glycerol, ketones, β-hydroxybutyrate, and acetylcarnitine concentrations increased. Pyruvate, lactate, alanine, and glutamate concentrations also increased. Plasma concentrations of valine (a BCAA) declined (*p* = 0.002) while valine degradation byproducts increased in association with decreases in urea cycle amino acids arginine and ornithine. Metrics of cardiopulmonary function were associated with increases in propionylcarnitine (C3, *p* = 0.013) and Ci4-DC/C4-DC (*p* < 0.01), byproducts of BCAA catabolism.

**Conclusions:** Induction of lipolysis, FAO, gluconeogenesis, and glycogenolysis provides critical substrates for cardiopulmonary function during exercise. However, none of those pathways were significantly associated with metrics of cardiopulmonary function. The associations between rVO_2_, and aVO_2_/HRmax and C3 and Ci4-DC/C4-DC suggest that the cardiopulmonary response to maximal exercise in adolescents is linked to BCAA utilization and catabolism.

## Introduction

Acute aerobic exercise induces anaerobic glycolysis, lipolysis, fatty acid oxidation (FAO), and glycogenolysis/gluconeogenesis to provide energy for cardiopulmonary function and maintenance of blood glucose for insulin-independent tissues like brain, red blood cells, and renal medulla (1). These adaptations are mediated by increases in cortisol, growth hormone (GH), catecholamines, and are facilitated by a decline in insulin (2–4).

Metabolic and physiologic responses to acute aerobic exercise have been well-studied in adults (1, 5), but poorly described in childhood and adolescence. While similarities no doubt exist, unique metabolic and physiologic responses are likely, given the dramatic cardiovascular growth and development that occur during puberty. Left ventricular (LV) mass and cardiac output increase during puberty and in response to aerobic exercise training (6, 7). Cardiac growth during puberty and cardiomyocyte hypertrophy following physical training might be linked by GH, which is induced in both puberty and acute exercise (8, 9). Growth hormone-induced cardiac growth with puberty and acute exercise may occur through several, not mutually exclusive pathways. Growth hormone increases during puberty might promote cardiac growth through induction of IGF-1 (10). Also, given that long-chain fatty acids are major energy substrates for the heart (11–13), the lipolytic effects of GH may increase cardiac contractility and LV mass by inducing increases in free fatty acids during exercise and aerobic training. The rise in GH after exercise is thought to be mediated by an increase in circulating catecholamines (14). However, conflicting results have been reported regarding the acute effects of exercise on ghrelin, GH, and the IGF-1 axis. Ghrelin is a GH secretagog in resting state (15), yet its role in the rise of GH secretion during or following exercise is unclear (16). In parallel with our analysis of changes in cortisol, insulin, and fatty acid metabolites, we determined if the rise in GH following exercise is preceded by, or associated with, a rise in ghrelin.

In addition to fatty acids, the BCAA might also serve as substrates for cardiac metabolism and growth, thus contributing to adolescent-specific metabolic and physiologic exercise responses. Studies in rodents, transgenic mouse models, and human adults suggest an increase in branched-chain amino acid (BCAA) catabolism during exercise (17–22). Moreover, branched-chain α-ketoacids (BCKAs), the products of BCAA transamination, are preferentially reaminated and promote protein synthesis in the heart (23). However, the relationship between BCAA catabolism and adolescent cardiopulmonary function has not yet been explored. To address some of these gaps in knowledge, we investigated the relationship between BCAA catabolism and metrics of cardiopulmonary function in healthy, well-developed, mature adolescent athletes undergoing an acute bout of maximal aerobic exercise.

We hypothesized that: (a) acute maximal exercise in adolescents would induce lipolysis, FAO, and BCAA catabolism in association with increases in GH and cortisol and a reduction in insulin; (b) the rise in GH would correlate with, and may be preceded by, an increase in ghrelin; and (c) metrics of cardiopulmonary function (aVO_2_, rVO2, and aVO_2_/HRmax) during maximal exercise would correlate with increases in GH secretion, FAO, and BCAA catabolism. To test these hypotheses, we obtained blood samples prior to and 30 min after the onset of maximal exercise testing and analyzed the levels of various hormones and metabolites by targeted metabolomic profiling, principal components analysis (PCA), and multiple linear regression models. This type of study—to the best of our knowledge—has never been conducted in adolescents. Given the limited data in this age group, our findings provide new insight into the physiology of aerobic exercise in adolescents.

## Materials and Methods

### Study Cohort

We recruited 11 sexually mature high-school students ages 14–18 years (7 males, 4 females) from two schools in the Durham area; all of whom were well-trained athletes participating in track and/or cross-country. The students were asked to perform maximal cardiopulmonary exercise tests under controlled conditions with a ramped protocol designed such that the entire exercise period lasted approximately 10 min. Female participants were tested for pregnancy. All participants were healthy and free of cardiovascular disease (including an arrhythmia) and were able to complete a maximal exercise treadmill test. Exclusion criteria included a history of diabetes or hypertension. Study subjects were asked to consume a meal containing protein and fruit 2–3 h before the maximal treadmill test, though the macronutrient content of this meal was not standardized.

### Informed Consent

This study was approved by the Duke University Institutional Review Board. Written informed consent was obtained from parents of children under 18. Written assent was obtained from children under 18 and informed consent was obtained directly from those who were 18 years of age.

### Blood Sampling and Analysis

Blood samples (6 ml) were collected prior to exercise (baseline) and 30 min after onset of maximal exercise at a time when GH levels were expected to peak (2). Samples were collected on ice and were treated with aprotinin (500 KIU/per ml blood) to prevent enzymatic protein degradation. EDTA plasma was stored at −80°C and analyzed at the Duke Molecular Physiology Institute.

Assays for conventional metabolites were performed using a Beckman DxC 600 clinical analyzer. These included measurements for glucose and lactate (reagents from Beckman, Brea, CA); total ketones, β-Hydroxybutyrate, and non-esterified fatty acids (NEFA) (Wako, Mountain View, CA); glycerol (reagents modified from triglycerides-blanked assay by Roche, Indianapolis, IN); and pyruvate (reagents from Sigma, St. Louis, MO for assessment of NADH disappearance). Insulin was measured by an electrochemiluminescent immunoassay performed using an SI-2400 imager and reagents from Meso Scale Discovery (Rockville, MD). Other immunoassays were performed on a Molecular Devices M2e plate reader (Mountain View, CA) using commercial kits for insulin-like growth factor-1 (IGF-1), GH, and cortisol from Alpco (Salem, NH); ghrelin from Millipore (Bilerica, MD); and glucagon from Mercodia (Uppsala, Sweden).

Acylcarnitines and amino acids were analyzed using stable isotope dilution techniques. The stable isotope labeled amino acids and acylcarnitines were used as internal standards. Ion ratios of metabolites to respective internal standards were converted to concentrations using external calibration curves constructed from authentic aliphatic acylcarnitines and amino acids (24, 25). The measurements were made by flow injection tandem mass spectrometry using sample preparation methods described previously (24, 25). The data were acquired using a Waters triple quadrupole detector equipped with Acquity^TM^ UPLC system and controlled by MassLynx 4.1 software platform (Waters, Milford, MA).

3-Hydroxyisobutyric acid (3-HIB) was analyzed by LC-MS/MS. Fifty microliters of plasma containing an isotopically labeled internal standard d6-2-hydroxyisobutyric acid (2-HIB) (CDN Isotopes) were precipitated with 400 μl of methanol. The methanol supernatants were dried, reconstituted in water, and injected onto a Waters Acquity UPLC system coupled to a Waters Xevo TQ-S triple quadrupole mass spectrometer. The analytical column (Waters Acquity UPLC HSS T3 Column, 1.8 μm, 2.1 × 100 mm) was used at 30°C; 10 μl of the sample were injected onto the column and eluted isocratically at 95% eluent A (0.1% formic acid in water) and 5% eluent B (acetonitrile) and a flow rate of 0.4 ml/min. The total run time was 6.5 min. Mass transitions of m/z 103 → 73 (3-HIB) and 109 → 62 (d6-2-HIB) were monitored in a negative ion electrospray ionization mode.

3-Amino Isobutyric Acid (BAIBA) was analyzed by LC-MS/MS. Fifty microliters of plasma containing an isotopically labeled internal standard d3-BAIBA (Medical Isotopes) were precipitated with 400 μl of methanol. The methanol supernatants were dried and esterified with acidified butanol for 15 min at 65°C. The samples were reconstituted in 5% methanol and injected onto a Waters Acquity UPLC system coupled to a Waters Xevo TQ-S triple quadrupole mass spectrometer. The analytical column (Waters Acquity UPLC HSS T3 Column, 1.8 μm, 2.1 × 100 mm) was used at 30°C; 10 μl of the sample were injected onto the column and eluted at a flow rate of 0.4 ml/min. The gradient began with 95% eluent A (0.1% formic acid in water) and was then programmed as follows: 0–4 min—gradient to 20% eluent B (acetonitrile); 4–6 min gradient to 90% B; 6–7 min—hold at 90% B, return to 95% A, and re-equilibrate the column at initial conditions for 1 min. Mass transitions of m/z 160 → 86 (BAIBA) and 163 → 89 (d3-BAIBA) were monitored in a positive ion electrospray ionization mode.

### Maximal Cardiopulmonary Exercise Test

**Figure 1:**
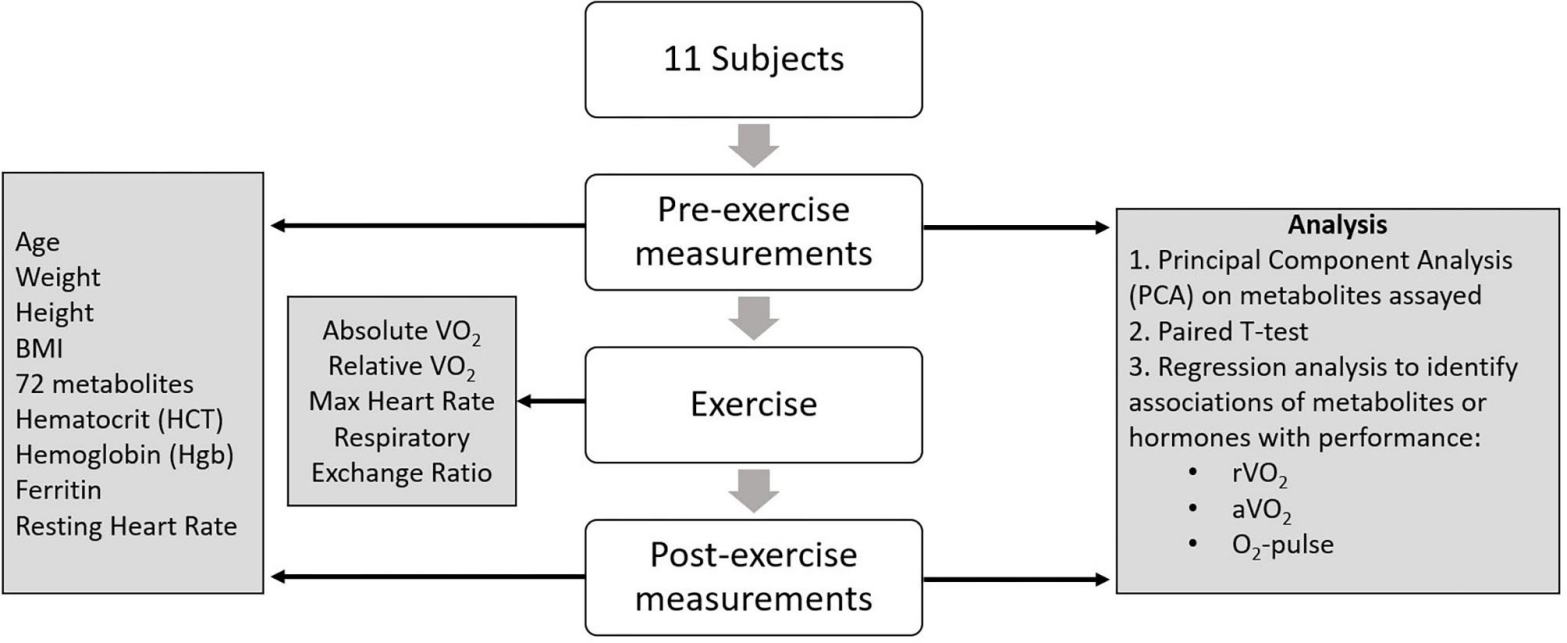
Study design.

Each study participant completed a maximal cardiopulmonary aerobic exercise test with 12-lead ECG and expired gas analysis on a treadmill. A standardized running protocol was used for all subjects, with 1-min incremental workload increases until volitional fatigue. Expired gases were analyzed continuously using a ParvoMedics TrueMax 2400 system (Sandy, UT) and values obtained during the final 30 s of each test were averaged to determine VO_2_ peak. Progressive exercise testing—i.e., driven by increasing workload, as in this case—relies initially and predominantly on slow muscle—fatty acid metabolism. As the workload increases, more fast—glycolytic—fibers are recruited, and therefore whole-body metabolism shifts from fat to glucose predominance. This is reflected in the progression of the RQ or RER from 0.80 (mixture of fat, protein and glucose) to 1.00 (glucose) to >1.0 (anaerobic metabolism) at the end of the test (26, 27). Subjects achieved an average peak respiratory exchange ratio of 1.18. Relative VO_2_ was expressed as ml/min/kg body weight. Due to the pilot nature of this study, we did not obtain direct echocardiographic measures of cardiac performance. Rather we used indirect metrics of cardiopulmonary function including peak relative (r), and absolute (a) VO_2_, and O_2_-pulse (aVO_2_/HRmax). Peak VO_2_ is a measure of cardiorespiratory capacity, while O_2_-pulse is a correlate of cardiac stroke volume (SV) per the Fick equation: aVO_2_ = HR × SV × A-VO_2_ diff (arteriolar-venous O_2_ extraction) (28). Figure 1 summarizes the study design.

### Statistical Analysis

Pre- and post-exercise metabolite data were log_10_ transformed to account for both normal and non-normal distributions among 72 metabolites. Paired, two-tailed student's *t*-tests were conducted on each metabolite to identify significant changes following exercise. Metabolites were assessed in all athletes, with analyses of sexes combined, as well as individually to identify both exercise-induced changes in metabolites and sex-specific exercise-induced changes. Although this was an exploratory pilot study, we calculated power using the pwr package in R (29); this revealed that we were well-powered to identify changes in a single metabolite level at a significance level of α = 0.05 (*d* = 1.59, *n* = 11, *p* = 0.99). Considering the number of metabolites assayed, we used the Bonferroni adjusted *p*-value of 6.9 × 10^−4^ (0.05/72) to define statistical significance. *P*-values ≤ 0.01 were considered nominally significant. We then calculated the Spearman rank correlation between the changes (pre- vs. post-exercise) in metabolites and hormone levels. Spearman rank correlation coefficients (rho) were plotted using a correlogram and hierarchical clustering via the corrplot package in R to visualize correlations between the change in metabolites and change in hormones of interest (GH, ghrelin, and cortisol) (30). Principal component analysis (PCA) was conducted using all pre- and post-exercise metabolite data to identify covariates for use in regression analyses. Metabolites with more than 25% of values below the lower limit of detection were excluded from analysis. Linear regression was used to determine if measures of cardiopulmonary function [aVO_2_, rVO_2_, and O_2_-pulse (aVO_2_/HRmax)] were associated with: (a) pre-exercise metabolite concentrations; and (b) changes in metabolite concentrations. To control for any effects or differences due to sex or school of origin, we included sex and school as covariates in all regression analysis. All analyses were conducted using R version 4.0.4 (2021-02-15) (31).

## Results

### Baseline Anthropometric and Performance Characteristics

Baseline anthropometric and performance characteristics stratified by sex are presented in Table 1. Eleven subjects—four females, and seven males—were studied. Each subject had a normal BMI; none were underweight or overweight (Tables 2A,B). Having attained their mid-parental target heights, they all were well-developed, mature adolescents. As expected, males had greater hemoglobin concentrations and hematocrits than did females. Resting and maximum heart rates, and rVO_2_ were comparable among males and females.

**Table 1:** Phenotype summary of the study cohort, baseline anthropometric, and performance characteristics.

**Sex**	**Females (*n* = 4)**	**Males (*n* = 7)**	***P*-value**
Age (years)	15.82 (±1.19)	16.19 (±0.78)	0.604
Height (cm)	164.97 (±7.14)	173.16 (±4.70)	0.101
Weight (kg)	50.75 (±6.64)	61.47 (±5.75)	0.038
BMI	18.94 (±0.8)	18.62 (±0.35)	0.487
BSA	1.54 (±0.13)	1.73 (±0.09)	0.052
HCT	40.08 (±0.96)	43.96 (±2.03)	0.002
Hgb	13.65 (±0.31)	15.01 (±0.72)	0.002
Ferritin	50.75 (±25.04)	101.14 (±38.61)	0.028
rVO_2_ (mL/kg/min)	58.3 (±4.98)	65.1 (±9.33)	0.150
aVO_2_ (L/min)	2.96 (±0.46)	4.02 (±0.81)	0.021
RER	1.16 (±0.06)	1.19 (±0.06)	0.412
TTE (s)	661.25 (±54.9)	755.57 (±53.43)	0.031
Rest HR (bpm)	58.33 (±5.51)	56.33 (±8.62)	0.755
Max HR (bpm)	194 (±9.7)	196.43 (±7.57)	0.683

**Table 2:** **(A)** Sex, age, weight, height, and BMI distribution of the study participants and **(B)** distribution of BMI percentiles of study participants.

**Sex**	**Age (yrs.)**	**Height (cm)**	**Weight (kg)**	**BMI**	**BMI %ile**
**(A)**					
M	15.47	168.91	53.2	18.65	26.5
M	15.72	168.91	66	23.13	79.9
M	15.34	181.356	70	21.28	65.9
M	15.86	172.72	59.5	19.94	42.6
M	16.75	177.292	59.2	18.83	17.6
M	17.31	169.672	64.9	22.54	64.2
M	16.90	173.228	57.5	19.16	20.7
F	14.93	158.75	41.8	16.59	7.3
F	15.15	164.846	51.1	18.80	33.2
F	15.66	175.006	57.8	18.87	30.4
F	17.55	161.29	52.3	20.10	36.3
			**Boys**	**Girls**	**Total**
**(B) SUMMARY OF CHILDREN's BMI-FOR-AGE**					
**Number of children assessed**			7	4	11
Underweight (<5th %ile)			0%	0%	0%
Normal BMI (5th−85th %ile)			100%	100%	100%
Overweight (≥85th %ile)*			0%	0%	0%
Obese (≥95th %ile)*			0%	0%	0%

* *Terminology based on Barlow SE and the Expert Committee (32)*.

### Pre- vs. Post-exercise Metabolites and Hormones

**Figure 2:**
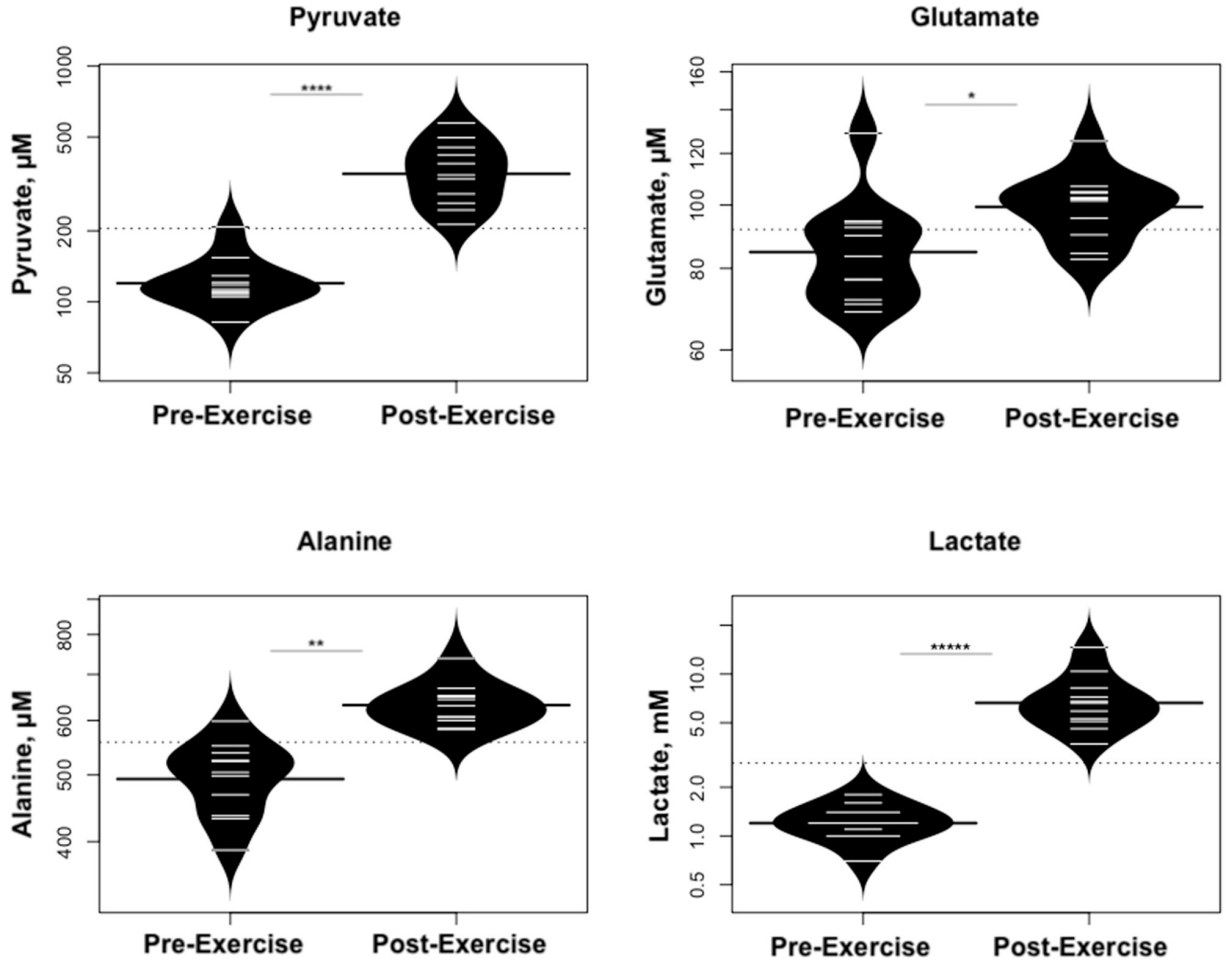
Changes in metabolites related to interplay between the skeletal muscle and liver in maintaining blood glucose levels and disposition of the nitrogen waste through the urea cycle (Cori and Cahill Cycles). *p < 0.01, **p < 0.001, ****p < 0.00001, *****p < 0.000001.

**Figure 3:**
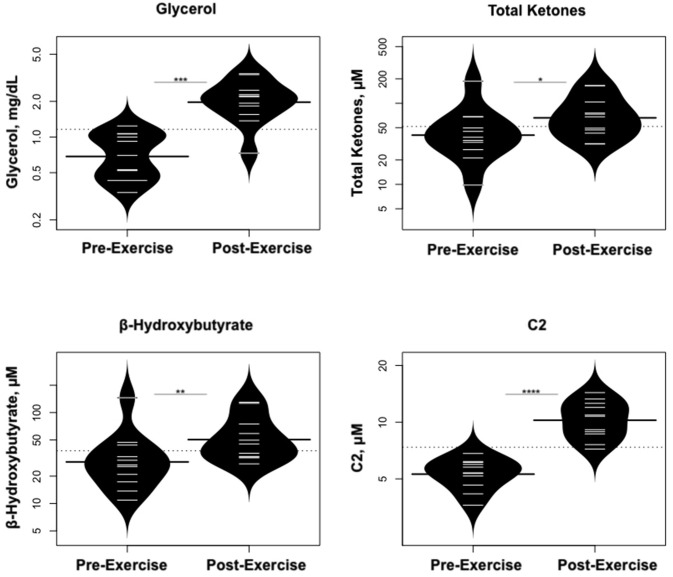
Changes in metabolites related to lipolysis/FAO. *p < 0.01, **p < 0.001, ***p < 0.0001, ****p < 0.00001.

**Figure 4:**
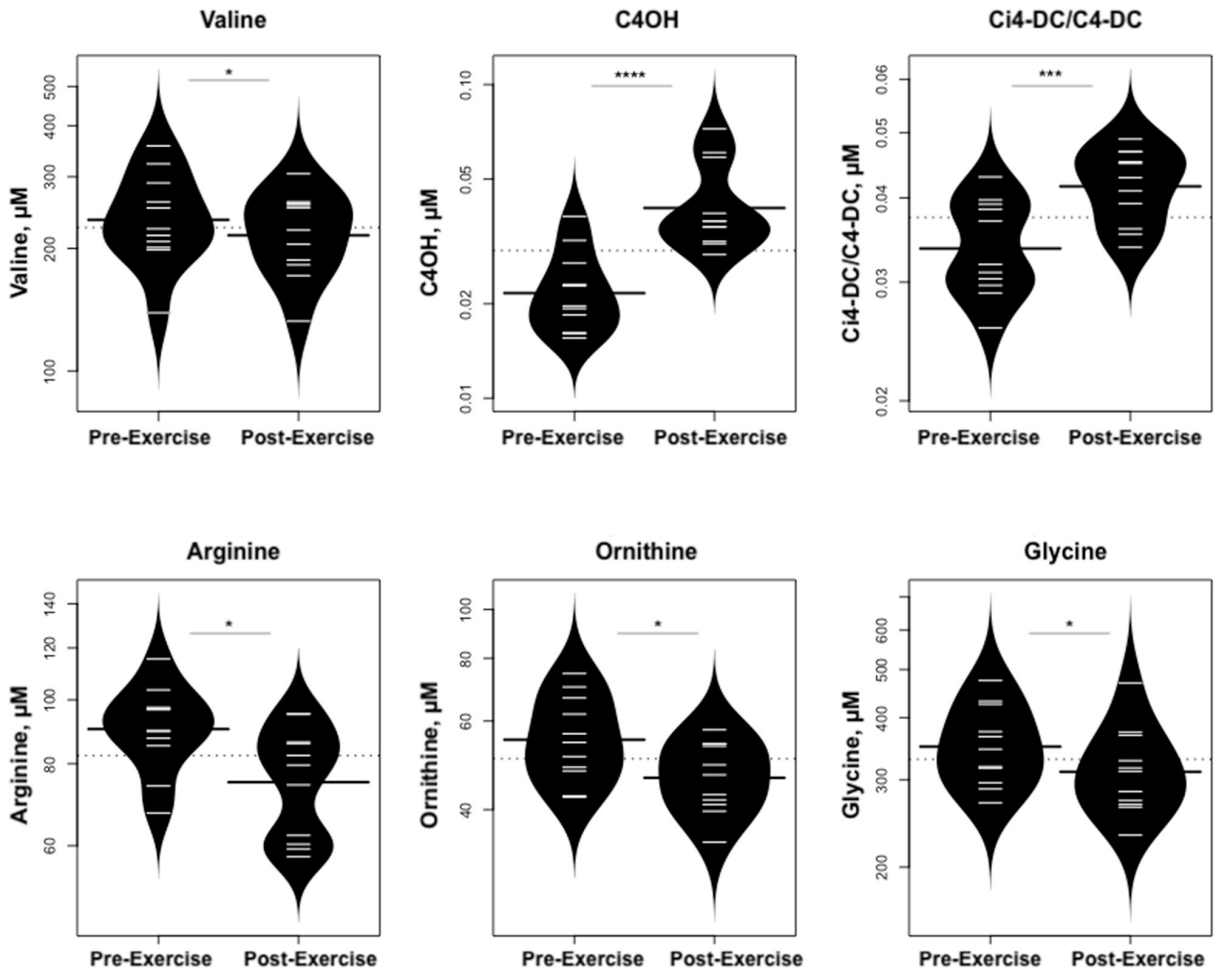
Changes in metabolites related to BCAA catabolism and urea cycle. *p < 0.01, ***p < 0.0001, ****p < 0.00001.

Statistically significant differences in metabolites and hormones pre- vs. post-exercise are listed in Table 3. Following maximal exercise, there were increases in pyruvate, glutamate, alanine, and lactate (Figure 2). Suggesting induction of lipolysis and FAO, the levels of glycerol, total ketones, β-Hydroxybutyrate, and acetylcarnitine (C2) rose (Figure 3). Consistent with increases in complete FAO, the concentrations of medium-chain acylcarnitines (C8, C10, C10:1, C12:1) fell. Concentrations of valine (a BCAA) declined, while valine degradation byproducts including 3-hydroxybutyrylcarnitine (C4-OH) and methylmalonyl/succinyl carnitine (Ci4-DC/C4-DC) increased, in association with decreases in the urea cycle amino acids arginine and ornithine (Figure 4). Of note, glycine declined following exercise (see section Discussion) (Table 3; Figure 4).

**Table 3:** Changes in hormones and metabolites in response to acute maximal aerobic exercise.

		**All**	**All**	**All**	**Males**	**Males**	**Males**	**Females**	**Females**	**Females**
		***P*-value**	**Pre-ex mean (SD)**	**Post-ex mean (SD)**	***P*-value**	**Pre-ex mean (SD)**	**Post-ex mean (SD)**	***P*-value**	**Pre-ex mean (SD)**	**Post-ex mean (SD)**
Changes in metabolites related to Cori and Cahill Cycles	Lactate, mM	200E-07	1.236 (0.304)	7.127 (3.085)	1.111E-04	1.314 (0.302)	8.029 (3.551)	3.429E-03	1.1 (0.294)	5.55 (1.139)
	Pyruvate, μM	560E-06	123.364 (33.092)	364.727 (112.875)	2.211E-04	128.857 (41.293)	397.857 (116.922)	9.642E-03	113.75 (6.801)	306.75 (89.916)
	Alanine, μM	820E-05	496.641 (60.793)	632.925 (45.334)	4.142E-04	492.622 (48.399)	620.444 (27.239)	4.440E-02	503.672 (86.781)	654.767 (66.085)
	Glutamate, μM	3.660E-04	86.117 (17.152)	99.999 (11.889)	7.377E-03	85.524 (20.63)	99.086 (14.896)	5.617E-02	87.153 (11.275)	101.597 (4.698)
Changes in metabolites related to lipolysis/FAO	C2, μM	390E-07	5.38 (0.945)	10.484 (2.374)	600E-05	5.749 (0.71)	11.82 (1.808)	3.581E-03	4.734 (1.045)	8.147 (0.897)
	C8, μM	3.707E-04	0.087 (0.024)	0.059 (0.016)	1.964E-02	0.088 (0.027)	0.063 (0.016)	7.753E-03	0.086 (0.021)	0.051 (0.014)
	C8:1-DC, μM	2.028E-03	0.028 (0.006)	0.026 (0.007)	1.996E02	0.03 (0.007)	0.027 (0.008)	9.385E-02	0.026 (0.005)	0.024 (0.006)
	C10, μM	240E-05	0.167 (0.064)	0.103 (0.033)	3.617E-03	0.157 (0.066)	0.103 (0.035)	1.449E-03	0.186 (0.065)	0.103 (0.036)
	C10:1, μM	7.645E-04	0.112 (0.029)	0.081 (0.025)	2.549E-02	0.121 (0.031)	0.092 (0.023)	1.666E-02	0.098 (0.02)	0.062 (0.019)
	C12:1, μM	1.493E-03	0.062 (0.019)	0.044 (0.012)	3.133E-02	0.061 (0.022)	0.047 (0.013)	3.540E-02	0.064 (0.016)	0.041 (0.011)
	Glycerol, mg/dL	140E-05	0.749 (0.317)	2.124 (0.796)	8.453E-04	0.689 (0.338)	2.319 (0.923)	1.357E-02	0.855 (0.29)	1.782 (0.406)
	β-Hydroxybutyrate, μM	9.603E-04	37.645 (37.576)	58.645 (37.134)	8.316E-03	47.071 (45.103)	73.986 (39.208)	1.044E-01	21.15 (8.242)	31.8 (3.455)
	Total ketones, μM	2.604E-03	52.873 (47.966)	77.527 (48.184)	1.668E-02	65.086 (57.48)	97.486 (50.596)	7.155E-02	31.5 (10.209)	42.6 (7.934)
Changes in hormones	Growth hormone, ng/mL	1.084E-04	2.984 (4.523)	15.957 (9.881)	3.565E-03	3.853 (5.603)	19.124 (10.746)	2.487E-03	1.462 (0.737)	10.415 (5.488)
	Total ghrelin, pg/mL	2.214E-04	387.909 (121.244)	317.091 (88.308)	8.643E-03	368.714 (113.639)	302 (87.539)	2.001E-02	421.5 (144.251)	343.5 (95.953)
	Cortisol, μM	2.607E-03	21.173 (6.722)	30.345 (4.537)	3.774E-02	23.429 (7.136)	31.543 (4.55)	6.061E-02	17.225 (4.019)	28.25 (4.245)
Changes in metabolites related to BCAA catabolism and urea cycle	C4-OH, μM	230E-06	0.023 (0.007)	0.042 (0.015)	6.795E-04	0.025 (0.008)	0.048 (0.015)	1.243E-03	0.018 (0.002)	0.032 (0.003)
	Ci4-DC/C4-DC, μM	6.560E-04	0.034 (0.006)	0.042 (0.005)	3.010E-02	0.036 (0.005)	0.042 (0.005)	6.986E-03	0.032 (0.006)	0.041 (0.006)
	Glycine, μM	1.059E-03	355.196 (66.366)	317.14 (67.062)	9.530E-03	336.104 (53.892)	292.798 (44.176)	5.329E-02	388.607 (80.832)	359.74 (85.356)
	Ornithine, μM	1.249E-03	56.064 (11.009)	46.834 (7.43)	1.119E-02	56.33 (10.423)	46.01 (6.124)	9.387E-02	55.596 (13.646)	48.275 (10.23)
	Valine, μM	2.387E-03	242.418 (62.38)	220.875 (50.234)	8.541E-03	254.669 (75.857)	227.774 (58.029)	2.090E-01	220.978 (22.344)	208.802 (37.03)
	Arginine, μM	5.588E-03	91.187 (13.282)	76.195 (14.287)	1.548E-02	85.717 (11.331)	71.666 (11.774)	2.181E-01	100.76 (11.8)	84.12 (16.479)

*C4-OH, 3-hydroxybutyrylcarnitine or beta-hydroxybutyrylcarnitine; Ci4-DC/C4-DC, methylmalonyl carnitine or succinyl carnitine; C8, octanoyl carnitine; C8:1-DC, octenedioylcarnitine; C10, decanoyl carnitine; C10:1, decenoyl carnitine; C12:1, dodecenoyl carnitine. Statistically significant differences in metabolites and hormones pre- vs. post-exercise are listed in red (p < =0.01)*.

**Figure 5:**
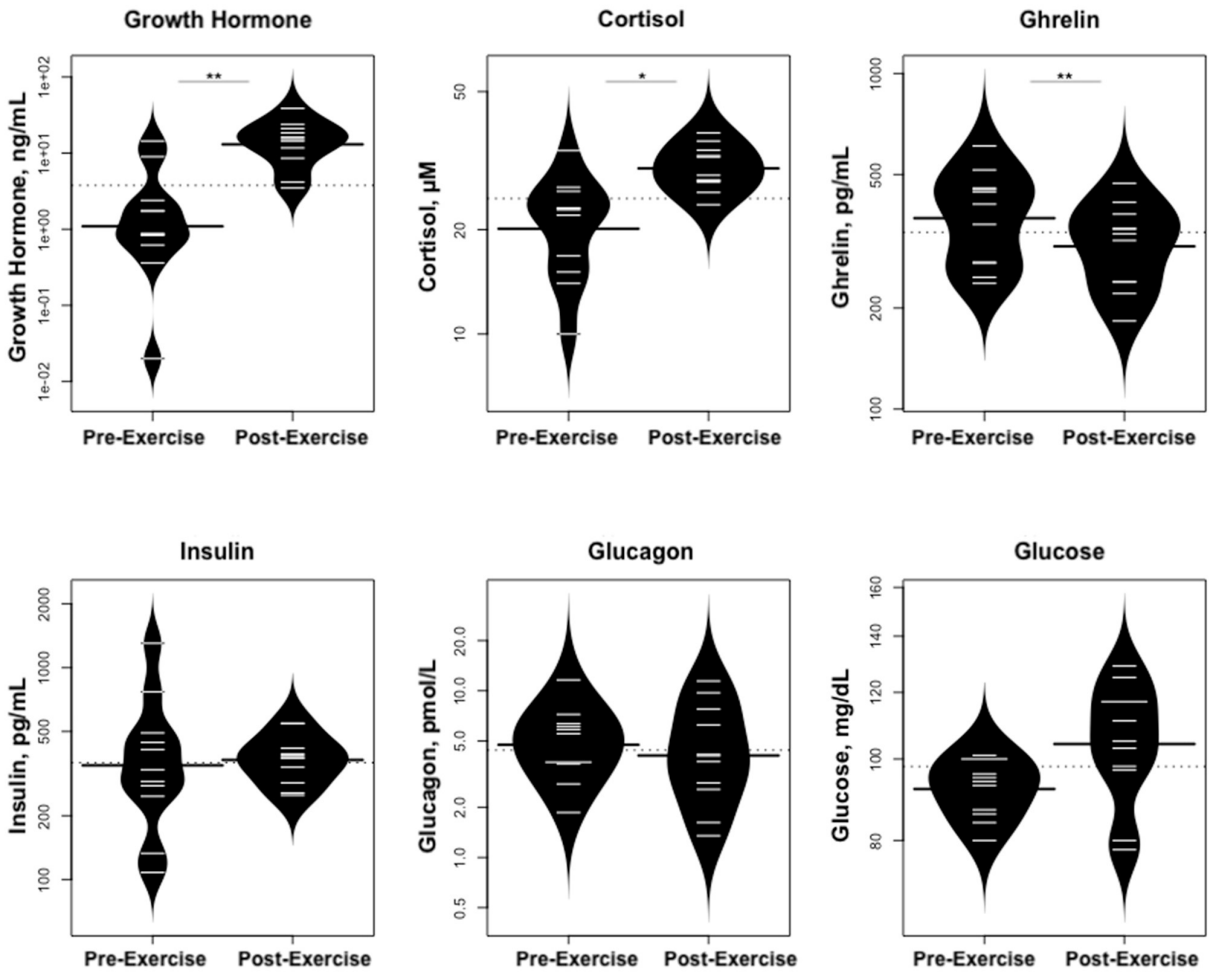
Changes in hormones controlling changes in metabolites and changes in glucose concentrations. *p < 0.01, **p < 0.001.

As predicted, GH increased dramatically following exercise (Figure 5), while IGF-1 and IGFBP-3 levels did not change; cortisol levels also rose. In contrast, levels of ghrelin declined. Glucose concentrations were stable, while there were no changes in glucagon or insulin concentrations.

**Figure 6:**
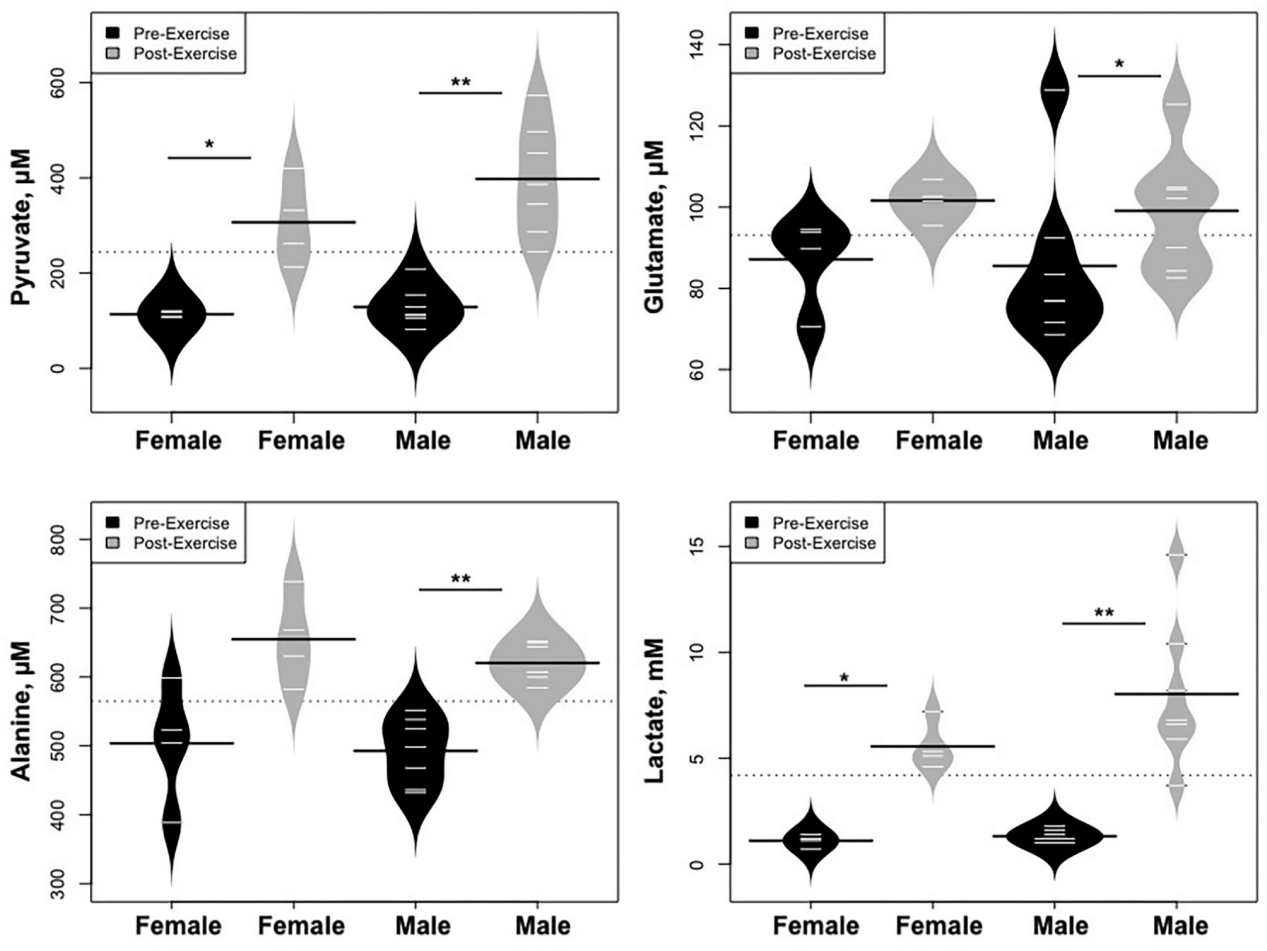
Changes in metabolites related to interplay between the skeletal muscle and liver in maintaining blood glucose levels and disposition of the nitrogen waste through the urea cycle (Cori and Cahill Cycles), stratified by sex. *p < 0.01, **p < 0.001.

**Figure 7:**
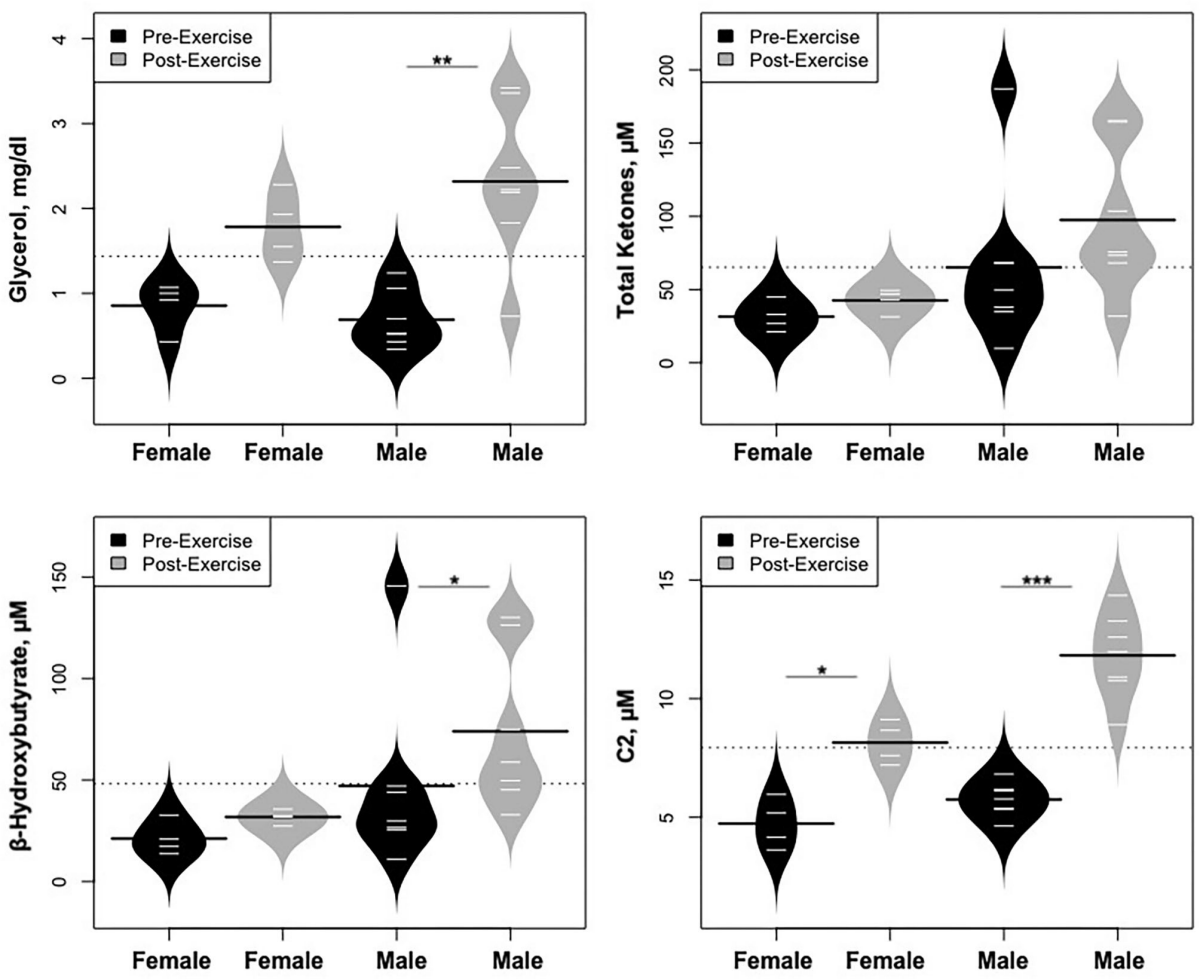
Changes in metabolites related to lipolysis/FAO, stratified by sex. *p < 0.01, **p < 0.001, ***p < 0.0001.

**Figure 8:**
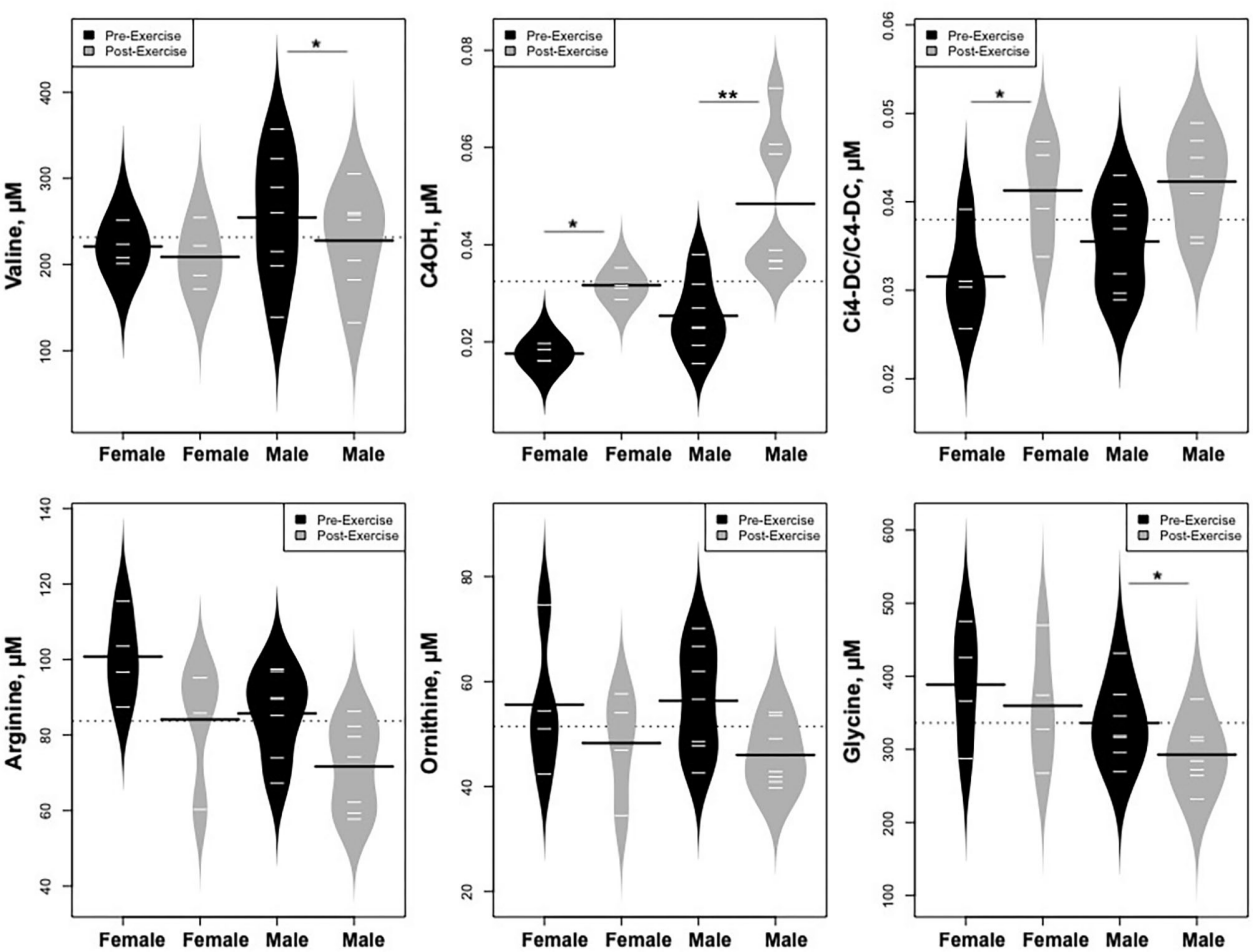
Changes in metabolites related to BCAA catabolism and urea cycle, stratified by sex. *p < 0.01, **p < 0.001.

**Figure 9:**
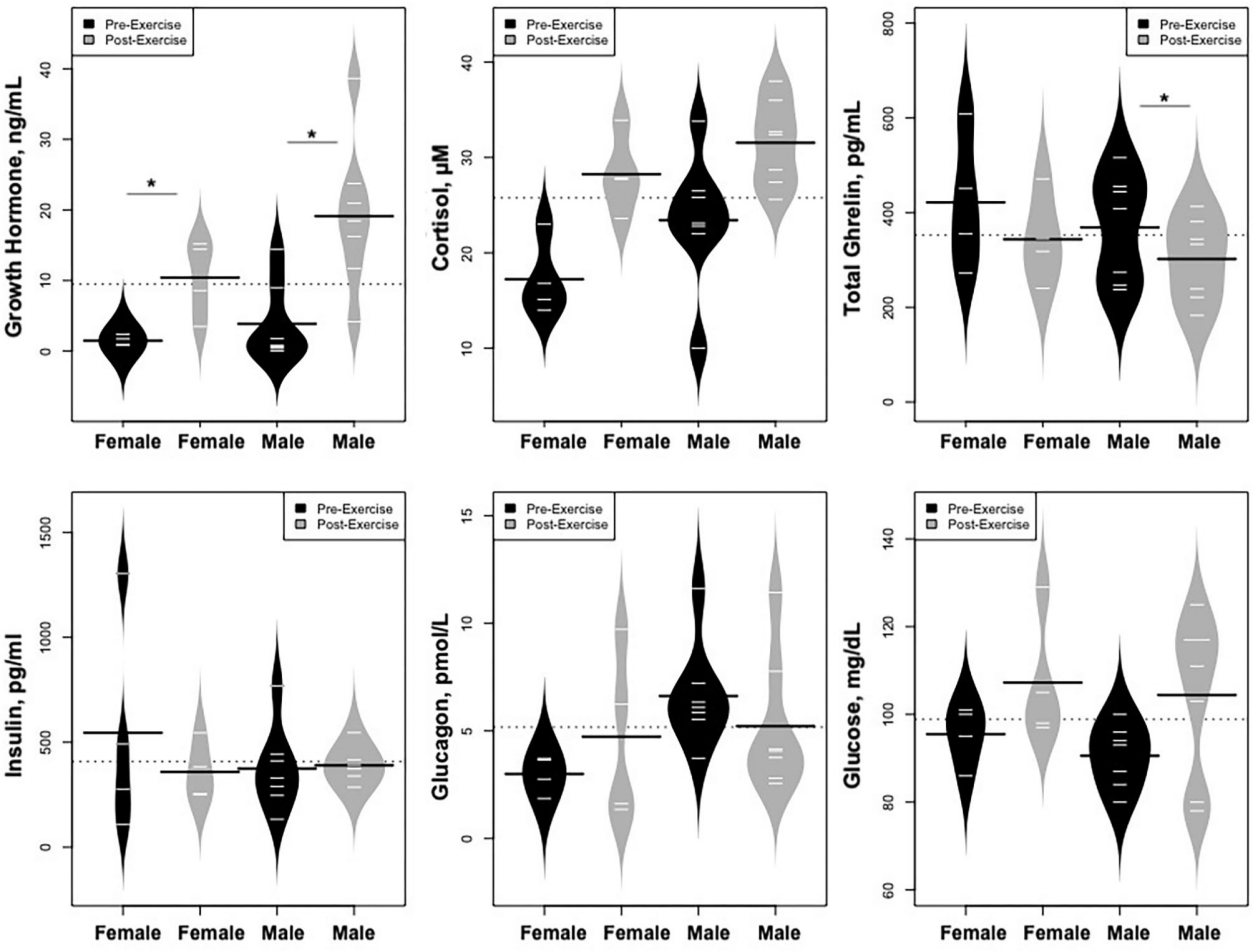
Changes in hormones controlling changes in metabolites and changes in glucose concentrations, *p < 0.01.

Statistically significant differences in metabolites and hormones pre- vs. post-exercise were stratified by sex; the results are shown in Table 3 and Figures 6–9. Both males and females had increases in GH, lactate, pyruvate, C2, and C4-OH levels following exercise. Only the males showed changes in alanine, glutamate, valine, glycine, β-Hydroxybutyrate, glycerol, and ghrelin. Conversely, only the females had a significant increase in the valine catabolite Ci4-DC/C4-DC, and a significant reduction in C8. Given that we had only four females in our cohort, the sex differences might be driven in part by the smaller number of female participants.

### Correlations Among Changes in GH, Cortisol, and Ghrelin and Changes in Metabolites

**Figure 10:**
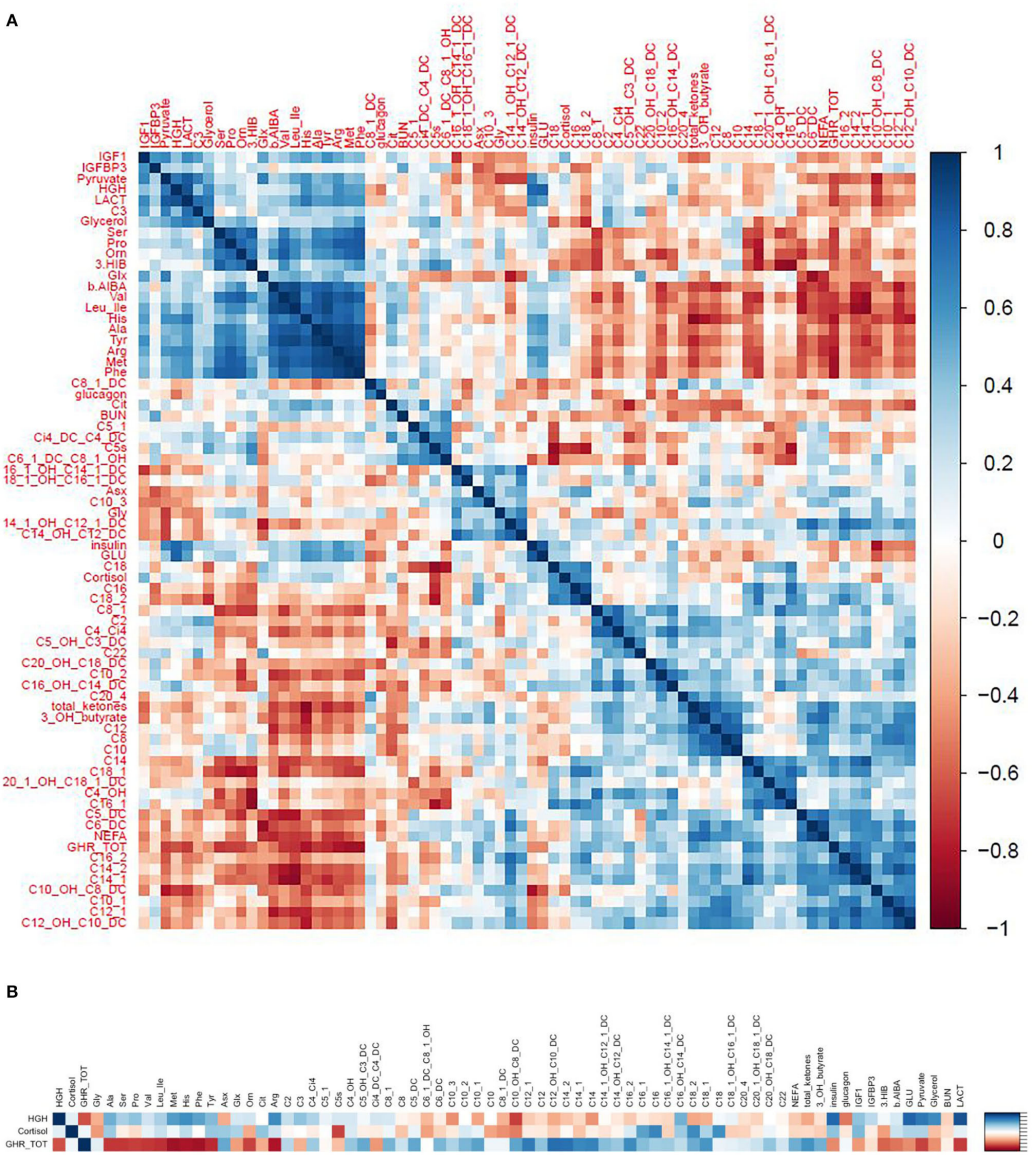
**(A)** Correlograms depicting the correlations (spearman rho) between changes in hormones and metabolites in response to exercise. **(B)** A focused view showing just the correlations between key hormones of interest (GH, ghrelin, and cortisol) and the most significant metabolic adaptions in response to exercise.

We used correlograms to depict the associations between changes in all hormones and metabolites (Figure 10A), and a more focused view (Figure 10B) showing just the associations for changes in key hormones of interest (GH, ghrelin, and cortisol) against the most significant metabolic adaptations in response to exercise.

#### Growth Hormone

Changes in GH correlated positively with changes in pyruvate, lactate, glycerol, and glucose and negatively with changes in medium to long chain acylcarnitines. These observations support the notion that GH induces lipolysis and counteracts the effects of insulin to maintain blood glucose levels in response to exercise (Figures 10A,B).

#### Ghrelin

We hypothesized that the rise in GH following exercise would be associated with, or preceded by, a rise in ghrelin. However, the change in ghrelin correlated negatively with change in GH. In fact, the relationships between change in ghrelin and change in GH and changes in various metabolites were completely in opposite directions, forming a mirror image (see the correlogram, Figure 10A). Change in ghrelin had strong negative associations with changes in BCAA (valine, leucine/isoleucine) and their catabolic byproducts HIB and BAIBA, urea cycle AA (ornithine, citrulline, arginine), aromatic AA (phenylalanine, tyrosine), and other AA (histidine, methionine, alanine, serine, proline) as well as pyruvate, lactate, glycerol, and glucose. In contrast, change in ghrelin correlated positively with change in medium to long chain acylcarnitines, NEFA, total ketones, and β-Hydroxybutyrate (Figures 10A,B).

#### Cortisol

Change in cortisol was strongly associated with changes in C5. Otherwise, associations with other metabolites were similar but of lesser magnitude than changes in GH (Figures 10A,B).

### Principal Components Analysis

**Figure 11:**
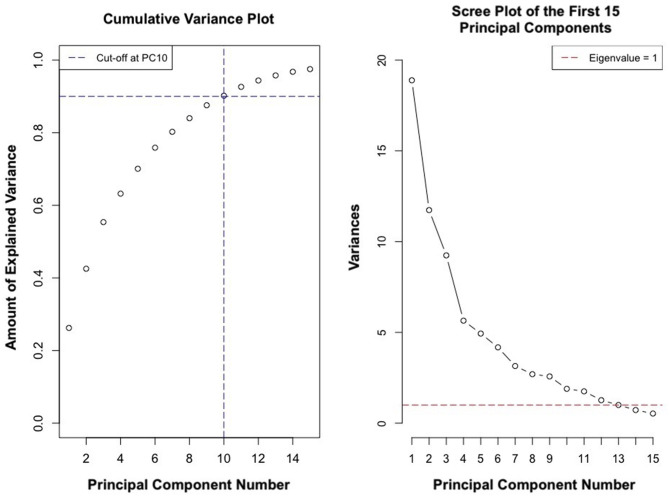
Cumulative variance and scree plot of the first 15 principal components.

PCs 1–12 had an eigenvalue of <1 (Figure 11). PCs 1–9 explained approximately 89% of the variance in the data (Figure 11). Analysis of PC weights failed to reveal variables that specifically defined each PC. Thus, principal components served to identify key covariates. PC1 was significantly different in athletes from different schools (*p* < 0.004) and PC2 was significantly different in males and females (*p* < 0.007). Sex and school were included as covariates in all regression analyses.

### Association With Performance

We analyzed the relationships between metrics of cardiopulmonary function (aVO_2_, rVO_2_, and VO_2_-pulse) and the pre-, post-, and changes in all metabolites and hormones measured in our study. Exercise-induced changes in Ci4-DC/C4-DC, a byproduct of BCAA catabolism, was associated with rVO_2_ (*R*^2^ = 0.75, *p* = 0.005) and O_2_-pulse (aVO_2_/HRmax) (*R*^2^ = 0.76, *p* = 0.01). Likewise, change in propionylcarnitine, a byproduct of BCAA catabolism, associated nominally (*R*^2^ = 0.67, *p* = 0.013 and *R*^2^ = 0.75, *p* = 0.013) with rVO_2_ and O_2_-pulse (aVO_2_/HRmax), respectively. Changes in IGF-1 (*R*^2^ = 0.71, *p* = 0.008) was also associated with rVO_2_. Change in 3-hydroxy-eicosanoyl carnitine (also called octadecanedioyl carnitine, C20-OH/C18-DC), a long-chain acylcarnitine, was associated with aVO_2_ (*R*^2^ = 0.79, *p* = 0.009); however it is difficult to interpret this latter association because the levels of this metabolite in circulation are extraordinarily low.

No other metabolites or hormones, or changes in those metabolites or hormones, were associated with metrics of cardiac performance.

## Discussion

Acute aerobic exercise induces metabolic responses including anaerobic glycolysis, lipolysis, FAO, and glycogenolysis/gluconeogenesis to provide energy for cardiopulmonary function and maintenance of blood glucose. These adaptations are mediated by increases in GH, cortisol, and catecholamines and facilitated by a decline in insulin. Studies in rodents, transgenic mouse models, and human adults suggest that BCAA also undergo catabolism during intense aerobic exercise (17–22). However, the relationship between BCAA catabolism and cardiopulmonary function has not yet been explored in humans. Here, we used targeted metabolomic profiling to assess exercise- induced changes in BCAA and their catabolic byproducts and their relationship to metrics of cardiopulmonary function including rVO_2_, and aVO_2_/HR max (O_2_-pulse).

Our findings include four important observations: (1) acute maximal aerobic exercise in adolescents induces anaerobic glycolysis, lipolysis, FAO, and BCAA catabolism to provide critical substrates for cardiopulmonary function; (2) the rise in GH following exercise is not associated with or preceded by a rise in ghrelin; indeed, ghrelin levels correlate negatively with GH; (3) changes in ghrelin following exercise correlate negatively with changes in BCAA and their catabolic byproducts and positively with metrics of lipolysis and FAO, while changes in GH correlate most strongly with metrics of carbohydrate metabolism; and (4) metrics of cardiopulmonary function (aVO_2_, rVO_2_, and O_2_-pulse) following maximal exercise associate most strongly with metrics of BCAA catabolism (Ci4-DC/C4-DC and C3), as well as changes in IGF-1.

Our findings provide new insight into the physiology of aerobic exercise and the interplay between skeletal muscle, liver, adipose tissue, and cardiac metabolism in adolescent males and females. As predicted, there were increases in lactate, pyruvate, alanine, and metrics of lipolysis and FAO, providing substrates for energy production and gluconeogenesis. Increases in complete FAO resulted in depletion of medium-chain acylcarnitines such as C8, C10, C10:1, C12:1, and increased concentrations of the end product acetylcarnitine (C2). Increase in C2 could also reflect increases in glucose oxidation, as studies have suggested that pyruvate dehydrogenase-derived acetyl CoA is more likely to siphon toward C2 via the actions of carnitine acetyltransferase, whereas FAO derived acetyl CoA is more likely to enter the Krebs Cycle (33). Lipolysis is likely driven in part by the rise in GH and catecholamines and, possibly, the fall in ghrelin (see below). Fatty acids are postulated to provide critical energy for the contracting heart and may enhance long-term cardiac growth during training (11–13).

Concentrations of the BCAA valine declined following the acute bout of aerobic exercise while the concentrations of its degradation byproducts 3-hydroxybutyrylcarnitine (C4-OH) and methylmalonyl/succinyl carnitine (Ci4-DC/C4-DC) increased, in association with decreases in urea cycle amino acids arginine and ornithine. Likewise, glycine declined. These findings signify that aerobic exercise triggers an increase in BCAA catabolism.

Branched-chain amino acid catabolism is initiated by branched-chain aminotransferase (BCAT), which facilitates a reversible transamination reaction generating BCKAs. This reaction involves conversion of α-ketoglutarate to glutamate (34). Excess glutamate in turn serves as a source of ammonia for generation of citrulline from ornithine in the urea cycle (35, 36). Thus, it follows that an increase in BCAA catabolism results in increased flux of nitrogen through the urea cycle and reduces levels of the urea cycle amino acids arginine and ornithine. Glycine serves as a carbon donor for the pyruvate-alanine cycle to dispose of excess ammonium generated by BCAA transamination (37).

The results of our studies are consistent with previous findings in experimental animals. For example, exercise capacity and intermediary metabolism were previously examined in skeletal muscle of mice with a genetic knockout of mitochondrial branched-chain aminotransferase (BCATm). Mitochondrial branched-chain aminotransferase KO mice were exercise intolerant with markedly decreased endurance to exhaustion. Thus, disruption of BCAA metabolism in mice impairs exercise metabolism and endurance (19). Studies in rats selectively bred for high running capacity suggest that efficient fatty acid and BCAA utilization contribute to high intrinsic exercise capacity (20). It was also reported that endurance exercise activates the BCKDH complex in rat and human skeletal muscles (21, 22).

Exercise is a well-established stimulus for GH secretion. The rise in GH following exercise in our study was not triggered by an increase in ghrelin, baseline hypoglycemia, or by low levels of IGF-1, all of which can stimulate GH secretion in fasting or malnutrition (38). Plasma ghrelin concentrations fell as GH concentrations rose. Nor was GH induced by a rise in plasma arginine, which at pharmacologic concentrations can promote GH secretion (39). It should be noted that we did not measure catecholamines, which rise in response to intense aerobic activity and stimulate GH secretion (40).

Interestingly, the decline in ghrelin following exercise was associated with increases in NEFA, total ketones, and β-Hydroxybutyrate (Figures 10A,B), suggesting that hypoghrelinemia might facilitate lipolysis and FAO. As ghrelin is associated with a reduction in blood pressure and heart rate in some studies; the reduction in ghrelin might also serve to optimize cardiac output in response to physical activity (41). Finally, the fall in ghrelin may serve to prevent maladaptive activities—such as food consumption—during times of intense physical exertion (42, 43).

The decline in ghrelin seen in our cohort is consistent with the response observed in a previous study in adults (4). However, the mechanism for this response is not well-understood. Ghrelin secretion is under the influence of a complex network of inputs, some of which may be altered during intense exercise. Certain inputs—such as activation of beta1-adrenergic receptors—may stimulate ghrelin secretion, while others—such as a rise in lactate and butyrate—may inhibit its secretion (42). In many studies, fasting and post-prandial ghrelin concentrations correlate inversely with insulin (43); in our study, change in ghrelin concentrations correlated negatively with change in insulin during exercise.

Despite multiple metabolic adaptations occurring in response to acute maximal exercise, the metabolic signature correlating most strongly with cardiopulmonary function comprised metrics of valine utilization and catabolism and IGF-1. This signature suggests a role for BCAA catabolism in the cardiopulomonary response to acute aerobic activity. Recent experimental data in rodents support this notion: BCKAs, are preferentially reaminated and activate protein synthesis in the heart (23). Thus, in addition to providing energy substrate for short-term cardiac activity, BCAA catabolism might play an important role in physiologic cardiac growth.

Physiologic cardiac growth in puberty is essential for peak cardiopulmonary development and performance later in life (6–9). Given that GH might promote physiologic cardiac growth through induction of IGF-1, the association between changes in IGF-1 and rVO_2_ warrants further investigation of the role of GH/IGF-1 axis in cardiopulmonary function in adolescents (9). Exercise-induced alterations of IGF-I seem to depend on several factors, including exercise type and intensity as well as the blood sampling protocol used in the study (16). In our study IGF-1 and IGFBP-3 levels did not change in response to acute exercise, consistent with earlier studies in pre-pubertal and pubertal children (9, 44, 45). It remains to be seen how chronic training in children over time will affect GH/IGF-1 axis and cardiac performance.

There were some limitations to our investigation. Due to the pilot nature of the study, we could not time the testing to the estrous cycle in female participants. However, pregnancy tests were negative and none of the adolescent females had unexplained amenorrhea. The metabolic differences between adolescent females and males might be explained in part by the small number of female subjects enrolled in the study, thus we cannot make definitive conclusions regarding exercise dependent metabolic differences between the sexes. We did not obtain direct echocardiographic measures of cardiac performance. Rather we used metrics of cardiopulmonary function including aVO_2_, rVO_2_, and O_2_-pulse (aVO_2_/HRmax). We did not have control over the training exposure prior to entry into the study, though most participants trained year-round. While we attempted to standardize food intake prior to blood sampling, there was likely some degree of inter-subject variability in macronutrient intake prior to each study.

Nevertheless, to our knowledge, this is the first study to use targeted metabolomics to explore changes in BCAA catabolism and determinants of cardiopulmonary response among healthy, well-developed, mature adolescents in response to acute maximal aerobic exercise. This study provides unique insights into the role of BCAA utilization and catabolism—a much-ignored fuel for energy metabolism and cardiac performance during aerobic exercise. Future longitudinal studies with larger cohorts are needed to identify differences during various stages of puberty; to differentiate the role of training from the effects of pubertal development; and to assess sexual dimorphism underpinning performance in adolescence.

## Data Availability Statement

The raw data supporting the conclusions of this article will be made available by the authors, without undue reservation.

## Ethics Statement

The studies involving human participants were reviewed and approved by Duke IRB. Written informed consent to participate in this study was provided by the participants' legal guardian/next of kin.

## Author's Note

The work was conducted at Duke University Medical Center, Durham, NC, United States.

## Author Contributions

MM and OI performed metabolomics and biochemical analysis and contributed to interpretation of the metabolomics data. PGB was responsible for interpretation of the data and writing the manuscript. MR performed advanced statistical analysis. KM, KH, and JJ contributed to data collection, database generation, and writing the manuscript. MF and WK were responsible for development of the research question, conception and design of the research project, interpretation of the data, and critical review of the manuscript. WK was responsible for funding of the project. All authors contributed to the article and approved the submitted version.

## Conflict of Interest

PGB receives support from Diabetes Research Connection, but has no relevant disclosures to this manuscript. MF is a co-investigator on a grant from the American Heart Association that deals with the pathogenesis and treatment of childhood obesity. MF is also the local PI on a Rhythm-sponsored study of identification and treatment of children and adults with monogenic obesity, and member of a Data Safety Monitoring Board for a separate Rhythm-sponsored study of treatment of patients with syndromic obesity. KM receives support from the Cystic Fibrosis Foundation, but has no relevant disclosures to this manuscript. The remaining authors declare that the research was conducted in the absence of any commercial or financial relationships that could be construed as a potential conflict of interest.

## Publisher's Note

All claims expressed in this article are solely those of the authors and do not necessarily represent those of their affiliated organizations, or those of the publisher, the editors and the reviewers. Any product that may be evaluated in this article, or claim that may be made by its manufacturer, is not guaranteed or endorsed by the publisher.

## Abbreviations

ALA: alanine
ARG: arginine
ASX: aspartate/asparagine
BCAAs: branched-chain amino acids
BCKAs: (branched-chain α-keto acids)
CIT: citrulline
CRP: C- reactive protein
GLX: glutamate/glutamine
GLY: glycine
hsCRP: high sensitivity C-reactive protein
HIS: histidine
KET: ketones
LACT: lactate
LEU/ILE: leucine/isoleucine
MET: methionine
ORN: ornithine
NEFA: non-esterified fatty acid
PHE: phenylalanine
PRO: proline
SER: serine
TYR: tyrosine
URIC: uric acid
C3: propionylcarnitine
C4-OH: 3-hydroxybutyrylcarnitine or beta-hydroxybutyrylcarnitine
Ci4-DC/C4-DC: methylmalonyl carnitine or succinyl carnitine
C8: octanoyl carnitine
C8:1-DC: octenedioylcarnitine
C10: decanoyl carnitine
C10:1: decenoyl carnitine
C12:1: dodecenoyl carnitine
C20-OH/C18-DC: 3-hydroxy-eicosanoyl carnitine/octadecanedioyl carnitine
aVO_2_: absolute total body oxygen consumption
rVO_2_: total body oxygen consumption
aVO_2_/HRmax: absolute total body oxygen consumption/maximum heart rate; O_2_-pulse.
